# A System of Surgery

**Published:** 1848-02

**Authors:** 


ART. III.—*A System of Surgery.* By J. M. Chelius, *public Professor of General and Opthalmic Surgery, Director of the Chirurgical and Opthalmic Clinic in the University of Heidelberg, &c., &c.* Translated from the German with Notes and Observations by John F. South, late Professor to the Royal College of Surgeons of England, and one of the Surgeon's to St. Thomas's Hospital; with additional Notes by G. W. Norris of Philadelphia, in three volumes, 8vo. Published at Philadelphia by Lea and Blanchard, 1847.

*Unnatural joints,* occur, says our author, from a variety of causes after fracture, among which advanced age, general disease, and especially too much motion, are the most common. Generally, if six months have elapsed since the fracture, and the fragments still move, a false joint may be considered as formed. They occur most frequently in the upper arm.

The treatment consists in rubbing the ends of the bone together, pressure with complete quiet; exposing and cauterizing the ends of the bone with caustic potash, nitric acid, butter of antimony, nitrate of silver, &c.; sawing off, or rasping the ends, and introduction of a seton. Of these, pressure and complete rest are most frequently successful. "When ablation is practised, it should be treated aftarwards" says Chelius, "as a compound fracture" and we are not to expect it to unite in a long time. I have known, however, at least one case in which the contrary practice proved successful: a false joint resulting from a fracture of the radius. The ulna had united shortened, and the radius overlaped without union. I exposed the radius and removed sufficient of both ends with a saw to reduce the fragments, and having thoroughly cleansed the wound, I brought it together *closely* with straps and roller, and sustained it with splints. In one week the wound was entirely healed, and in three weeks the bone was united. The seton, to be successful, must generally be kept in a long time, but it is less dangerous than ablation.

" *Hare-lip* can only be cured by operation. Although experience has shown that the operation may be successful in very young childred, it is, however, best to delay it till eight months, only when wolf's jaw is connected with hare-lip and the child cannot suck, may the operation be undertaken within the first six months. n children of two years the operation may be delayed till they eco me intelligent."

In a note we find the opinion of Lawrence, who recommends the operation as early as the third, fourth, or fifth month, and of Mutter, who operates upon children three, four and five days old ! While Mr. South himself, does not think even eight months sufficient age. He would never perform it under two years, and he prefers the sixth or eighth year. And we incline to the opinion of South, that there is actual danger in operating for hare-lip upon very young children, since we have heard from a gentleman, himself a witness, of convulsions and death immediately ensuing upon an operation when the child was only a few weeks old; and we have seen other similar cases recorded. There is no propriety, however, in deferring the operation beyond the age of two years, at least we do not think any necessity exists on the score of danger. while the delay to the fifth or sixth year is liable to the serious objection that the child having formed his habit of speaking, will never after so competely overcome a defective pronunciation as if the lip had been united earlier. The period between the second and third year of life, ought generally to be chosen.

*Cleft palate, torn perineum, ulcers, scalded head, and milk crust,* or *crusta lactea infantum,* constitute the subjects of the succeeding chapters.

*Itch, scabies,* or *psora,* has, since the advent of Homœopathy, acquired a rank and importance of which the humble *acarus scarabaei,* dwelling in his itch pustule, never dreamt. And, by-the-by, since the "itch mite" has become the cause of so large and respectable a class of maladies, why are they not admitted into the Homœopathic Pharmacopoeas? According to the maxim "*similia similibus curanter,*" an infusion of one of these microscopic gentry ought to constitute a sovereign remedy for all those ailments of which it has been discovered to be the cause.

Chelius says the cause of itch is a *contagious matter*, and he considers the part which the itch-mite may take in its production as yet undecided. Their existence, however, is not disputed at present. They are found chiefly between the fingers, and on the wrist, and they are "little wee bodies," with a trunk, proboscis, and six or eight very hairy feet, which are, under the magnifying glass, continually in motion. It is a nocturnal animal, only wandering about during the dark, which, according to Aube, explains why the disease is almost alone communicated by sleeping with an itch patient. The number of itch-mites has no direct proportion to the intensity of the disease; generally there are but few itch-mites on a person affected with the itch.

The most conclusive experiments which have been made to show that these mites actually may produce the disease in question were made in 1812, by Gales, apothecary to the Hospital St. Louis; according to whom a mite confined under a watch glass upon the hand soon penetrated the skin, and, in a few hours afterwards, three vesicles arose, attended by an intense itching.

"Simple itch is cured by any remedy which destroys the itch-pustule, and produces such inflammation in the skin that the cuticle mortifies and is thrown off. On this circumstance may be explained the great number of remedies, and modes of treatment recommended for the itch, which only operate when in their composition they have such substances as bring about the above mentioned effects, or are so energetically rubbed in, that the *rubbing itself* produces the same result."

Thus frequent soap and water ablutions are recommended, soap baths, flowers of sulphur with soap and water, grease alone, or grease with mercury, nitrate of ammonia, sulphate of zinc, hellebore, &c. &c. But the liniment which our author prefers is Horns liniment, composed of one part of flowers of sulphur and two parts of black soap, with as much water as may be necessary to make it fluid. This is to be rubbed in four times a day, to the amount of four or six drachms, and with so much force as to produce a burning sensation, and peel off the diseased skin; at the same time also warm baths are to be frequently used. South has fully tried this plan and finds it superior to any thing else.

"In the Belgian army liquid sulphuret of lime is used for the cure of the itch according to the following order:—" Each patient is to be supplied with an ounce or an ounce and a half of liquid sulphuret of lime in a small pot. This quantity he is to rub carefully and slowly with his hands on every part that is covered with papulæ. If there be any papulæ on the back, another patient is to rub the liquid on that part. The operation is to be repeated three times in the twenty-four hours so that each patient consumes three or four ounces of the sulphuret daily. A bath is to be taken every alternate day; the frictions are to be suspended on that day. Fifteen frictions (or ten day's use, are usually sufficient for the cure of the disease, if the medical officer in charge sees that the remedy is properly used. *Preparation of the sulphuret of lime.*—Take of sublimed sulphur 16 lbs. and of quick lime 32 lbs. and boil in 80 lbs. of water for three quarters of an hour. Let the mixture rest for some time until it settle, and then let the clear fluid be decanted off. Boil the residue afresh in about the same quantity of water, treat it in a similar manner, and add this decoction to the first. Usually 140 lbs. of the sulphuret, at 12° by the areometer are thus obtained. If the liquid be more dense, it should be lowered to this standard by tne addition of rainwater."

In the treatment of *Lues*, our author recommends general, as well as local treatment. The local treatment is applications of nitrate of silver, caustic potash, corrosive sublimate and nitrate of mercury. The latter I can personally recommend as superior even to the nitrate of silver in power and promptness of action—seldom requiring more than one or two applications. These applications are to be followed by unguents or astringent washes, and the ulcers must be kept clean. To this must be added a mild steady mercurial treatment carried to *moderate* ptyalism. Lawrence and South also, deem mercury given internally the best protection against secondary symptoms in nearly all forms of lues, phagedenic and sloughing sores, of course, excepted. Lawrence is of opinion that modern surgeons, among whom he must of course include M. Ricord and Chelius, use mercury too little, for although a slight action upon the gums is sometimes sufficient, yet, in other cases it is necessary to push it to more copious salivation; nor should the use of the mercury be suspended when the sore is only cicatrizing, but not entirely closed—indeed, it is well, he thinks, to continue it ten or fourteen days after the ulcer has headed. Green says the action of mercury should be continued, on an average, three weeks for a chancre, four weeks for a bubo, and six or eight weeks for secondary symptoms. Buboes are to be treated according as they are active or indolent, by bleeding, cold lotions, cold poultices, &c., or by gray mercurial ointment, mercurial and hemlock plaster, soft poultices, iodine ointment, &c.

"The violent dispersion of an idiopathic bubo may accelerate the outbreak of the general venereal disease." The practice therefore of compression suggested by Fricke, and the application of blisters, followed by a solution of sublimate, we presume does not find favor with Chelius.

The general symptoms are to be treated also by mercury in various forms, either internally or externally, and Mr. South thinks that while nodes may be made to disappear perhaps more rapidly under the use of iodide of potash than of mercury, yet the cure will not be found so permanent.

The *Friction-cure*, recommended by Louvrier and Rust, may be resorted to when other means fail. The patient being prepared by bathing, purging, diet, and sometimes bleeding, frictions with the gray ointment are employed. Commencing with one drachm, the quantity may be gradually increased to two drachms, to be rubbed in every second or third day; generally nine rubbings are necessary, sometimes only five, and twelve are nearly always sufficient.

*Caries.*—We apply the term caries to an unhealthy species of ulceration of the bone, where there is no reparative process, but the parts are in a process of destruction. It occurs mostly in spongy textures, and is probably always preceded by inflammation and softening.

The general treatment of caries must be various, according as the patient may be scrofulous, syphilitic, gouty, rheumatic, &c., &c. The local treatment consists in excluding the air from the sore and giving free vent to the matter. No stuffing with tents; nor generally does our author approve of injections of anykind, either mild or acrid. The mild are inefficient, and the acrid do harm. It is better to wash the sore externally with mild aromatic baths. In some cases we may excise the carious bone.

*Necrosis*, is death of the bone, and is easily distinguished from caries.

"Although *caries* and *necrosis* are alike in many symptoms, as in the two diseases similar causes give rise to both, as the bones are laid bare by the removal of the soft parts, and less or greater portion of the substance of the bone is lost and suppuration is present, yet they are distinguished by the following circumstances:—*Caries* occurs especially in bones of a spongy texture, *necrosis*, on the contrary, in bones of a closer character; in superficial *caries* the swelling has not at first so great extent as in bony gangrene; the swelling in *caries* mostly opens itself to a greater extent, fistulous passages often occur which become callous; in *necrosis* these openings have ordinarily a fleshy wall; in *caries* stinking *ichor* flows but in *necrosis* true pus, which is only bad when retained too long by improper treatment, or when the neighborhood is much irritated, or when *caries* also exists; in *caries* there are vital appearances, injection of vessels, loosening up, suppuration and successive destruction, therefore is the touch of the probe painful, whilst the necrosed bone is insensible and pain is only produced by harsh touching, and which spreads to the sound parts; the softening which accompanies or precedes *caries* extends further and loses itself imperceptibly, and above all, where it exists the bony cells are filled with reddish fluid; in *necrosis* it is developed in sound parts, is less extensive, and produces only one layer of granulations, which are formed between the healthy and dead parts; the fungosities of a carious bone are softer, grayer, more discolored than those above and below a piece of the necrosed bone, and the latter have more of the appearance of the granulations of a suppurating wound; in *caries* the bone is rough, uneven, soft, broken up, fungous, and the probe easily penetrates it; in *necrosis* the bone is even and hard, and though rough, not, however, yielding and soft ; the bony splinters thrown off are in *caries* small, dust-like, and destructible; in superficial *necrosis* they are layer-like, in deep *necrosis* large, firm, and of the natural condition of bone; the *periosteum* or medullary membrane is in *caries* usually much changed or destroyed, whilst the one or the other or even both in *necrosis* preserve their integrity, therefore nature does little in *caries;* in favorable cases the openings becomes callous, and similar changes are produced in the bony substance, or granulations arise from the bone which become connected with those from the soft parts but are rarely converted into bone and form only misshapen masses, whilst in *necrosis* a more or less complete reproduction of the destroyed bones is effected. The course of *necrosis* is mostly tedious, but often also quick and connected with active inflammation, which is rarely the case in *caries*. After opening the abscess, the pain in *necrosis* usually subsides, whilst in *caries* it mostly increases.

The notion that in *caries* the organic principle (bony gelatine) has entirely disappeared, and instead of it a peculiar fatty matter produced which fills the cells of the carious bone, whilst in *necrosis* the formative principle remains unchanged and its respective relations, that is as they are found in health, remain, (Delpech, Berard, Pouget and others,) is not confirmed by Mouret's experiments, as according to him the fatty matter in which every writer believed is always found in recent *caries*, well distinguished from rancid fat by its smell, which may serve as its characteristic; but all the bones he examined have a fibrous jelly-like substance, and contain a proportionate quantity of saline substances, as in healthy bone; it is therefore impossible by the chemical characters indicated to distinguish *caries* from *necrosis*."

The general treatment must be directed to the general indications. The local treatment must be mild and unirritating.

"All irritating treatment to promote the throwing off of the *sequester*, as the enlargment of the fistulous apertures, the application of sharp spirituous remedies, the actual cautery, the repeated boring of the dead piece of bone, are hurtful, inasmuch as by their effect on the living part they increase the destruction, but on the dead bone have no effect at all."

Excision and amputation may become necessary.

Under the subject of carious teeth, Mr. South speaks of *bleeding* after extraction of a tooth.

"The bleeding which ensues occasionally in consequence of drawing a tooth of a person who has hæmorrhagic *diathesis*, is a matter of very serious consequence. and has sometimes destroyed life. I have seen several severe, though not fatal cases of this kind, and as it seemed to me that the blood came from the very bottom of one or other of the fang-sockets, when the tooth had more than one, I presume that the bleeding vessel was the proper artery of the tooth, and that the difficulty in arresting the hæmorrhage depended on the difficulty of getting at the vessel itself; for unless it can be immediately acted upon, whatever be the local application, it is sure to fail. Various remedies have been proposed; I have tried most of them without success, but I have never failed with the actual cautery, when I have properly applied it, that is, when its point has been sufficiently small to descend into the very bottom of the fang-socket. When I first had recourse to this practice I failed from not thrusting the hot wire (which makes the best cautery for this purpose, and should not be more than a line thick) down to the very bottom of the socket; but having corrected this error, I have never failed since, in at least half-a-dozen cases; and; therefore, believe that its inefficiency in the hands of others has arisen from the same cause which at first failed me. There is nothing very frightful in the employment of this remedy, nor does it produce more than momentary and slight pain, and I think it is therefore, best to resort to it at once without wasting time, and allowing the patient to lose blood to such extent as to disturb the constitutional powers, and excite any latent phthisical tendency, of which I have known an instanee."

Various other remedies have, however, been recommended, and although we think, with Mr. South, that the actual cautery well applied is the most reliable, yet the other remedies may occasionally be found sufficient. Some have used caustic potash, but it is hazardous; nitrate of silver, has also been used successfully, and pieces of sponge have been saturated with nitric acid, sulphate of copper tannin, benzoic acid, tincture of myrrh, muriated tincture of iron, alcohol, &c. Some reinsert the tooth; Cortez introduces wax into the socket; Peter Cullen recommends a soft phial-cork gently pressed into the socket, and carrying before it a small piece of lint moistened with some styptic; Ryan relates a case cured by internal use of ergot.

*Dislocations.*—Mr. South remarks that he has seen one case in which the femur being thrown into the ischiatic notch, the limb remained quite movable several hours, and he thinks he has seen other similar cases in which the usual sign of dislocation, and *immobility* was not present.

Old and very young persons are not so liable to dislocation as the middle aged. Mr. Travers, however, saw a dislocation of the femur into the ischiatic notch in a boy five years old; but probably there are very few instances upon record of an accidental dislocation of the hip at this period of life. The following is the result of Malgaignes, investigations as to the relative frequency of different dislocations.

"Dislocations of

| | | | | | |
|---|---|---|---|---|---|
| the shoulder . | 321 | thumb . | 17 | knee . . | 7 |
| hip . | 34 | wrist . | 13 | spoke-bone | 4 |
| collar-bone. | 33 | fingers . | 7 | knee-cap. | 2 |
| elbow. . | 26 | jaw. . . | 7 | spine . . | 1 |
| foot. . . | 20 | | | | |

And also that from the age of two to fifteen years, dislocation of the shoulder occurred only once out of four dislocations, but after sixty years about once out of one and a-half."

"My friend Mr. Liston, tells me, that he once reduced, without assistance, a dislocation on the back of the hip-bone, two or three minutes after it had occurred, by the person having been thrown from his horse, simply by putting his hand on the *pelvis* and pulling and rotating the thigh with the other. This is probably an unexampled case, but it proves, that the earlier the reduction is attempted the less power have the muscles to offer resistance."

Here is a text for my friend, Mr. A., who reduces all dislocated thighs, *if* his own word may be taken, unaided, without either extension, or counter-extension, simply by a kind of *tour de maitre* by a peculiar, mysterious, *secret*, anatomico-physiologico-scientific grand barbers flourish! It is a precious pain-saving, people-humbugging, pocket-filling discovery, which Mr. South has not heard of, or he would not have spoken of Mr. Liston's case as "probably unexampled." With us *such* cases are sufficiently common.

The means for effecting extension and counter-extension, are the hands of assistants, pulleys, &c., &c.; and where the resistance is not easily overcome, we think that, in addition to the bleeding, antimony, &c., mentioned by Chelius, we may now safely add, inhalation of sulphuric ether, or of the still more modern lethiferous gas, *chloroform.* For the effects of ether in difficult cases of dislocation, we refer the reader to some reports in the preceding numbers of this journal.

"I have known an instance in which a dislocated upper arm after having been reduced and carefully bandaged up, was left undisturbed for some weeks, and on the removal of the bandages was found to have slipped out, and could not be reduced again."–*South.*

We quote this passage for the benefit of those legal gentlemen who, after a dislocation has occurred, finding the bone still out of place, can see no way to account for it except by supposing that it was never reduced. It was such logic as this which persuaded a jury in Livingston Co., a few years since to impose a most unjust fine upon a distinguished surgeon; not because it could be shown that he did not reduce the shoulder, bnt solely because the shoulder was not now reduced.

Several of the succeeding chapters are devoted to the subject of herniae in its various forms and is more complete than any thing we have seen upon the subject. Prolapse of the uterus, vagina, bladder, rectum, with inversion and retroversion of the uterus, succeed; also torticollis, curved spine, curved limbs, club-foot, contracted fingers, &c., &c. The division appropriated to aneurisms is fullr and includes a notice of the recent mode of operation by compression.

Naevus and erectile tumors generally, Chelius considers under the head of "Teleangiectasy," or unnatural expansion of the capillary-vascular system. Speaking of the danger of extirpation with the knife, Mr. South says:—

"I have known two instances in which children died upon the operating table in extirpating a teleangiectasy from the face; and at least in one of the cases, no one could doubt the capability of the highly-distinguished operator."

Where it is broad and superficial, especially in children Chelius prefers the caustic potash.

The following paragraph may be of value to some of our young men, or ladies, whose faces are afflicted with what Hopkins the "witch finder general" of England used to call the "devils mark" and "witch spots."

"The tattooing of moles on the skin, proposed by Pauli, has yet to be mentioned. The part should be washed with soap and water, and rubbed till the blood is introduced into the most delicate branches of the erectile tissue; the skin is then made tight, and covered with color similar to the skin, which is formed of white lead and carmine. Three needles, sunk into a cork pad so that their points project, are then thrust into the skin, and their points from time to time dipped into the paint. In extensive spots we must proceed gradually, so as not to produce too great swelling. The most difficult part is the choice of color corresponding to that of the skin."

Varices, varicocele and piles close the volume. Chelius prefers the knife to the ligature in extirpating piles, but as we think on insufficient grounds. F. H. H,

